# EDUCATION, ADULT LITERACY, AND HEALTH AMONG MIDDLE-AGED AND OLDER IMMIGRANTS IN THE UNITED STATES

**DOI:** 10.1093/geroni/igae098.2873

**Published:** 2024-12-31

**Authors:** Donnette Narine, Takashi Yamashita, Runcie Chidebe, Phyllis Cummins, Jenna Kramer, Rita Karam

**Affiliations:** University of Maryland Baltimore County, Baltimore, Maryland, United States; University of Maryland Baltimore County, Baltimore, Maryland, United States; Miami University, Oxford, Ohio, United States; Miami University, Oxford, Ohio, United States; RAND Corporation, Arlington, Virginia, United States; RAND Corporation, Santa Monica, California, United States

## Abstract

The education-health pathway is well-documented whereby greater human capital such as educational attainment can provide health benefits over the life course. However, there is evidence of diminishing health benefits from education for immigrants compared to non-immigrants, partially due to structural factors (e.g., health insurance) limiting health-related benefits among immigrant populations. Older immigrants are more likely to be college-educated compared to their non-immigrant counterparts. However, the decades-old degrees from their home countries, along with English not being their native language, pose a particular concern for older immigrants’ health. As formal educational attainment cannot be easily promoted among older populations, adult literacy proficiency is another useful form of foundational human capital indicator, which is malleable at any life stage. This study examined the mediation effect of adult literacy proficiency on the education-health pathway among middle-aged and older immigrants in the United States. The nationally representative sample of immigrants aged 45 years and older (n = 590) in the 2012/2014/2017 U.S. Program for International Assessment of Adult Competencies (PIAAC) restricted-use file (RUF) data were analyzed. The mediation analysis showed that the association between educational attainment (college vs. less than college degree) [95% Confidence-Interval (CI): Lower-Limit (LL), Upper-Limit (UL) = 0.168, 0.780] and health (good vs. fair/poor) was mediated by adult literacy proficiency scores (0 to 500 points) [95% CI: LL, UL = 0.053, 0.352] among older immigrants in the United States. Findings from this study are informative to future interventions to support the health of older immigrants by way of adult literacy training.

